# The impact of the COVID pandemic on the treatment of psychoactive drug addicts in Zenica-Doboj Canton of BiH april 2020-april 2021

**DOI:** 10.1192/j.eurpsy.2022.1322

**Published:** 2022-09-01

**Authors:** S. Kasper

**Affiliations:** Cantonal Institut of Addiction, Inpatient Department, Zenica, Bosnia and Herzegovina

## Abstract

**Introduction:**

The paper presents experiences in working with drug addicts in Ze-Do Canton after the outbreak of the COVID pandemic

**Objectives:**

The time frame in which the research was conducted was April 15, 2020 to April 15, 2021. Criteria for inclusion in the study were clinically and laboratory-proven dependence on psychoactive substances and participation in some of the types of treatment in our institution. Criteria for exclusion from the study due to population specificity were not defined

**Methods:**

. The study was designed as a retrospective-prospective in which the following parameters were monitored: rate of retention in treatment, rate of relapse and overdose, deterioration of basic psychopathology, number of hospitalizations due to worsening addiction or comorbid psychopathology, suicide rate, incidence and prevalence of blood-borne hepatitis and HIV -a, incidence and prevalence of COVID in the addicted population and auto and hetero-destructive behavior of health care users.

**Results:**

The results of the study indicated an increased rate of abuse of substitution therapy, an increased rate of relapse, most often with stimulants, abuse of sedatives, antidepressants and anticholinergics, an increased rate of overdose but no deaths and an increased rate of hospitalization due to worsening basic psychopathology.

**Conclusions:**

The study indicated a deterioration in the quality of health care of addicts to psychoactive substances caused by pandemic working conditions and a marked deterioration in basic psychopathology caused by social distancing and the impossibility of more frequent and direct contact with patients. continuous monitoring

**Disclosure:**

No significant relationships.

